# Improving Staff Wellbeing in a Community Mental Health Team: A Six-Week Mindfulness-Based Quality Improvement Project

**DOI:** 10.1192/bjo.2026.11407

**Published:** 2026-06-30

**Authors:** Helen Kay, Emmalene Fish

**Affiliations:** 1 Pennine Care NHS Foundation Trust, Manchester, United Kingdom; 2 Pennine Care NHS Foundation Trust, Many, United Kingdom

## Abstract

**Aims:**

Staff working in community mental health teams (CMHTs) experience high levels of occupational stress and burnout, which can impact wellbeing, staff retention, and quality of patient care. Mindfulness-based approaches have demonstrated benefit for stress reduction, but their implementation within routine psychiatric services for NHS staff remains limited.

Aims were to improve wellbeing and reduce perceived stress among CMHT staff through a brief, structured mindfulness-based wellbeing programme delivered as a quality improvement initiative.

**Methods:**

A six-week mindfulness and wellbeing course was delivered to CMHT staff within a routine clinical setting. The programme was designed and delivered in person by the lead author, a clinician with formal training in mindfulness and meditation, rather than via digital or pre-recorded materials, ensuring consistency of intervention delivery.

Sessions included psychoeducation, brief mindfulness practices, and practical stress-management strategies. Wellbeing was measured using the World Health Organization Five Well-Being Index (WHO-5) pre- and post-intervention. Perceived stress was assessed before and after each session using a 0–10 self-report scale. Optional qualitative feedback was collected via free-text comments to explore participants’ experiences of the intervention. All questionnaires were anonymised using unique participant codes, allowing responses to be linked longitudinally without identifiable information.

**Results:**

Post-intervention, WHO-5 scores demonstrated an improvement in self-reportedwellbeing after six weeks compared with baseline scores. Mean perceived stress scores also showed an overall reduction across individual weekly sessions. Qualitative feedback identified themes of increased calm, improved emotional regulation at work, and a greater sense of psychological safety within the team.

**Conclusion:**

This quality improvement project suggests that a brief, mindfulness-based intervention is feasible, engaging, and may improve wellbeing among CMHT staff. Such interventions may represent a low-cost, scalable approach to supporting staff wellbeing within psychiatric services. Further cycles with larger samples and longer follow-up are warranted.

